# Tolerability and Effects of 2-Aminoethyl Dihydrogen Phosphate in Dogs With Mast Cell Tumors

**DOI:** 10.3389/fvets.2022.898077

**Published:** 2022-07-12

**Authors:** Eric Vieira Januário, Samanta Rios Melo, Durvanei Augusto Maria, Carla Aparecida Batista Lorigados, Aline Magalhães Ambrósio, Marcia Mery Kogika, Bruno Cogliati, Helio Junji Shimozako, Julia Maria Matera

**Affiliations:** ^1^Department of Surgery, School of Veterinary Medicine and Animal Science, University of São Paulo (FMVZ-USP), São Paulo, Brazil; ^2^Laboratory of Development and Innovation, Institute Butantan, Butantan, São Paulo, Brazil; ^3^Department of Internal Medicine, School of Veterinary Medicine and Animal Science, University of São Paulo, São Paulo, Brazil; ^4^Department of Pathology, School of Veterinary Medicine and Animal Science, University of São Paulo, São Paulo, Brazil; ^5^Laboratory of Bovine Viruses, Biological Institute of São Paulo, São Paulo, Brazil

**Keywords:** canine, chemotherapy, antineoplastic phospholipids, immunohistochemistry, toxicity

## Abstract

Canine mast cell tumor is a malignant neoplasm, and a gold standard treatment remains to be determined despite the proposed chemotherapies or other therapies in dogs. This study aimed to determine therapeutic, adverse effects and toxicity, tumor-free, and overall survival times of 10 dogs with surgically excised mast cell tumors evaluated by histopathological/immunohistochemistry and treated with four weekly intravenous administrations of 2-Aminoethyl Dihydrogen Phosphate (70 mg/kg) as adjuvant therapy. No adverse events were noted. Laboratory changes were limited (*p* < 0.05) in red blood cell, hemoglobin, and platelet counts. Mean tumor-free and overall survival were 599.1 ± 469 and 755.5 ± 423.5 days, respectively. In conclusion, 2-Aminoethyl Dihydrogen Phosphate administration was safe in dogs. However, 2-Aminoethyl Dihydrogen Phosphate was not sufficiently effective to prevent a recurrence, new tumor, or metastasis of canine mast cell tumors with poor immunohistochemical prognostic factors.

## Introduction

Canine mast cell tumors (MCT) affect the skin and subcutaneous tissues and may be also found in other tissues and organs, such as lymph nodes, the gastrointestinal tract, the liver, and the spleen. The relevance of these tumors stems from their malignancy and metastatic potential, with significant impacts on quality of life and survival of affected dogs (1–4). Disease progression varies according to clinical stage and MCT histological grade (5–7).

Skin MCT may be classified into three types (Grade I, II, or III) based on histopathological features, or described as well or poorly differentiated (high and low grade, respectively) (5, 6). Subcutaneous MCT are classified as circumscribed, infiltrative or combined (8, 9). Anatomical location (10), breed (1, 11), local recurrence (12), systemic manifestations (13), genomic (14), c-kit mutation (15, 16), expression of cell proliferation and growth rate markers such as AgNor, PCNA, and Ki67 (16–18), microvascular density and mitotic index (19) are other prognostic factors involved in MCT clinical progression.

MCT treatment includes surgical resection, cryosurgery, chemotherapy and radiotherapy, alone or in combination (3). Chemotherapy with vinblastine (12, 20), lomustine (21), clorambucil (22) or other antineoplastics such as masitinib mesilate (23), and toceranib (20) may be combined with

glucocorticoids such as prednisone and prednisolone (20, 24). However, a gold standard treatment for MCT remains to be determined (20). The aforementioned drugs are not free of adverse effects and toxicity in dogs and their use has been associated with neutropenia (21, 25), gastrointestinal toxicity (21, 25), pyrexia (21, 25), liver toxicity (24, 25) and pancreatitis (25), among other problems.

The search for novel antitumor therapies led to the investigation of a new class of drugs: antineoplastic phospholipids. Phospholipids are antitumor analogs, such as 2-Aminoethyl Dihidrogen Phosphate (2-AEH2F), which is a phosphorylated compound capable of controlling cellular proliferation and inducing apoptosis in several types of tumor cells (26). One drug has been derived from 2-AEH2F, a phospholipid cell membranes substrate (27). Although its mechanism is not fully understood, it is demonstrated that its effects are dependent on the incorporation in the cell membrane, modifying its structure and leading to cell death. It has been proposed that the internalization of 2-AEH2F into the cell membrane occurs via endocytosis mediated by lipid rafts. Recent studies have demonstrated the crucial requirement of Phosphoethanolamine (PE) in regulating mitochondrial function, change morphology and important role in autophagy (28, 29). PE constitutes about 25% of mammalian phospholipids and in brain tissue it reaches 45% of this composition. PE plays an important role in contractile ring disassembly at the cleavage furrow during cytokinesis of mammalian cells and a lack of PE inhibits progression of the cell cycle; demonstrating its antiproliferative potential similar to 2-AEH2F (30, 31).

The phospholipid compound 2-AEH2F is cytotoxic for a variety of cell tumors, such as human melanoma (SK-MEL-28, MEWO), murine melanoma (B16-F10) (32), human leukemia cell (33), murine hepatocellular carcinoma (Hepa1c1c17) (34), human breast cancer (MCF-7) (35), Ehrlich ascitic tumor (36), and murine renal cell carcinoma (37). In dogs 2-AEH2F was recently tested as an intravenous neoadjuvant therapy for soft tissue sarcoma and promoted a decrease in peritumoral skin temperature, which suggested the physiological effects of the substance on this type of neoplasia (38).

Given the need to develop novel antineoplastic therapies targeting MCT, this study set out to determine the *in vivo* effects of four weekly intravenous administrations of 2-AEH2F soluble preparation in dogs with skin or subcutaneous MCT, to evaluate treatment effects on overall (OS) and tumor-free survival (TFS), and to investigate adverse events and laboratory changes in treated patients. The hypothesis is that 2-AEH2F may be effective in preventing canine MCT recurrence, new tumor growth or metastasis, aside its immunohistochemical classification.

## Materials and Methods

### Experimental Design

A small, single-arm, open-label, uncontrolled experimental research with 10 dogs diagnosed with skin or subcutaneous MCT. The study was not blinded with the objective that the dog owners knew and agreed with the instituted therapy. As it is a descriptive study on tolerability and efficacy, it was decided to describe such circumstances in a limited number of 10 dogs, because it is a sample size that we could achieve in our hospital.

All experiments were carried out in accordance with the Ethics Committee for the Use of Animals of the Faculty of Veterinary Medicine and Zootechnics of the University of São Paulo (FMVZ-USP, São Paulo - Brazil) (protocol number 9825270116). In the clinical trial, informed consent to participate in the study was obtained from the dog owners prior to any study procedure.

Inclusion criteria were the presence of one or more skin neoformations eligible for surgical removal, diagnosis of cutaneous or subcutaneous MCT by post-surgical histopathological examination. Exclusion criteria were dogs with previous tumor history, other concomitant neoplasms, MCT metastases, except in excisable lymph nodes, non-operable tumors, anesthetic, surgery, and chemotherapy contraindications.

### Animals and Tumors

Ten household dogs (7 males, 3 females) were seen at the Small Animal Surgery Department of the Veterinary Hospital (HOVET) of the School of Veterinary Medicine and Animal Science of the University of São Paulo (FMVZ-USP, São Paulo - Brazil) and diagnosed with skin or subcutaneous MCT. The median age of dogs was 9.5 years (range: 5–13 years) and the median weight was 15.9 kg (range: 1.7–39.2 kg). The dogs resided and fed freely with their owners. Dogs presented with skin or subcutaneous masses, erythematous (*n* = 5) or not (*n* = 7), sessile (*n* = 10), or pedunculated (*n* = 2) and no other clinical manifestations. The initial diagnosis was obtained using fine-needle aspiration using a 25-ga needle. The material collected was gently spread on glass slides, smeared and stained by Panoptic technique. All dogs underwent surgical excision of MCT. The tumors were excised with dogs under general inhalation anesthesia (isofluorane). A 3.0 cm safety margin for all sides and a fascial plane was removed. Surgical wound closure was performed with simple suture or advancement or transposition flaps. Excisional tissue specimens had diameters of 0.4–8.0 cm (median = 2.2 cm), soft (*n* = 7) or firm (*n* = 5), ulcerated (*n* = 2) or not (*n* = 10), irregular surface (*n* = 4) or smooth (*n* = 8), alopecic (*n* = 4) or not (*n* = 8), hyperpigmented (*n* = 3) or not (*n* = 9). The characteristics of the dogs and MCT are listed in Table 1.

**Table 1:** Signalment and tumor characteristics of dogs treated with 2-AEH2F.

**Dog**	**Breed**	**Age (years)**	**Sex**	**Tumor location**	**No. of tumors**	**HP classification**	**Metastasis at diagnosis**	**Margins**	**KIT staining pattern**	**Ki67 (%)**
1	MB	12	M	Scrotum	1	Grade II Low Grade	No	Deep margin compromisse	III	23.36
2	Golden Retriever	8	M	Prepuce	1	Grade II Low Grade	No	Free	II	15.27
3	MB	8	M	Scrotum	1	Grade II Low Grade	No	Free	No staining	1.96
4	Cocker Spaniel	9	F	Perianal	1	Grade III High Grade	No	Dermis obliteration by tumor	II	24.45
5	Golden Retriever	5	F	4th PL dígit	1	Grade II Low Grade	Popliteal lymph node	Free	I	5.71
6	Pinscher	11	F	PL	1	Grade II Low Grade	No	Deep margin compromisse	II	2.93
7	Pug	5	M	Skin (2 abdominal, 1 PL)	3	Grade II Low Grade	No	Free	I	8.01
8	MB	13	M	Scrotum	1	Grade II Low Grade	No	Free	I	14.12
9	MB	10	M	Abdominal region	1	Grade II Low Grade	No	Free	NP	NP
10	Labrador	12	M	Skin	1	Subcutaneous	No	Free	NP	NP

*2-AEH2F, 2-Aminoethyl Dihydrogen Phosphate; MB, mixed breed; PL, pelvic limb; NP, not performed*.

### Product

The 2-AEH2F, sterile solution (pH = 7.2) was synthesized by the Chemistry and Polymer Technology Laboratory of the University of São Paulo (USP- São Carlos, Brazil) and prepared to achieve 99% analytical purity, confirmed by high-performance liquid chromatography (HPLC). For *in vivo* trials, the 1M stock sterile solution is dissolved in water and stored at room temperature. Stability has been demonstrated elsewhere (33, 35–37).

### Histological and Immunohistochemical Analysis

Surgical specimens (*n* = 10) were stored in 10% formaldehyde, then embedded in paraffin, cut, and stained with hematoxylin and eosin for histological examination. Patnaik, Kiupel and Thompson classifications were used (5, 6, 9). Immunohistochemical analyses (kit immunostaining and Ki67 expression, *n* = 8) were also run for prognostic factor determination (Table 1).

### 2-AEH2F Therapy

Following surgical resection and histopathological confirmation of MCT diagnosis, patients (*n* = 10) were treated with four doses of intravenous 2-AEH2F (70 mg/kg diluted in sterile 0.9% saline solution) at weekly intervals (D1, D8, D15, and D22).

### Patient Examination and Follow-Up

Medical history collection and physical examination were carried out before surgery (D0), on drug delivery days (D1, D8, D15 e D22), and within 30 days of the last 2-AEH2F administration (D52). Changes detected on physical examination, tumor recurrence, and/or development of new tumors were recorded. Dogs were then reassessed every 90 days for 6 months and every 180 days thereafter. Tumor recurrence, new tumor growth, or metastasis development were investigated, and survival rates determined. At time points D0, D8, D15, D22, and D52 dogs were submitted to the following tests: complete blood count, kidney (urea and creatinine) and liver (alanine aminotransferase/ALT, alkaline phosphatase/AP and albumin) biochemical analysis, and fasting triglyceride, total cholesterol, and blood glucose level determination. Dogs were also submitted to eco and electrocardiographic assessment (D0) and transabdominal ultrasonography (D0, D52, then every 90 days for 6 months and every 180 days thereafter).

### Laboratory Tests

Laboratory tests were performed at HOVET-FMVZ-USP Clinical Laboratory. Blood samples were collected into dry and EDTA-containing tubes and immediately processed. Dry tube samples were spun at 2,500 rpm for 10 min for blood serum separation. Complete blood count was carried out using automated equipment (ADVIA 2120i - Siemens Healthcare Diagnostics Inc., Camberley, UK) and light microscopy. Liver and kidney biochemical analysis, blood glucose, triglyceride, and total cholesterol level determination were also performed using automated equipment (LabMax, Labtest, Tokyo, Japan). Laboratory result analysis was based on mean values and standard deviations obtained at D0, D8, D15, and D22. Adverse events secondary to chemotherapeutic and antineoplastic agents were also investigated according to the criteria of the Veterinary cooperative oncology group (39).

### Statistical Analysis

Dogs in this sample were assessed at five-time points (D0, D8, D15, D22, and D52). The following variables (20) were evaluated: red cell count, hemoglobin levels, packed cell volume, leukocyte count (band and segmented neutrophils, lymphocytes, monocytes, eosinophils, and basophils), platelet count, blood glucose, triglyceride, and total cholesterol levels, and serum ALT, AP, albumin, urea and creatinine levels. Measures of central tendency (means and medians) were calculated for each variable. Measures of dispersion (standard deviations) and upper and lower limits of 95% confidence intervals were calculated for respective means. Data were collected from the same dogs at each time point; therefore, samples were defined as a dependent.

The non-parametric Friedman test was used for variable-specific analysis at different time points, with the level of significance set at 5% (α = 0.05). The non-parametric Wilcoxon *post hoc* test was then applied with a Bonferroni-adjusted level of significance of 0.5% (αBonf. = 0.005). Overall and TFS data were used to construct Kaplan-Meier survival curves.

## Results

### Animals

This sample comprised 10 dogs with histological diagnosis of skin (*n* = 9) or subcutaneous (*n* = 1) MCT and free from cutaneous or sonographic evidence of metastasis on D0. Enlarged popliteal lymph node was noted only in one dog and metastasis histologically confirmed following surgical resection. MCT were surgically resected and histologically classified as per Patnaik and Kiupel (5, 6), as follows: grade II/low grade (GII/LG), *n* = 8; grade III/high grade (GIII/HG), *n* = 1; subcutaneous (SC), *n* = 1. Tumors were also described as having free or compromised surgical margins (*n* = 8 and *n* = 2, respectively). Eight histological samples were submitted to immunohistochemical analysis, whereas two samples were not amenable to this test due to the absence of adequate and enough histopathological material for this examination (Table 1).

### 2-AEH2F Treatment and Follow-Up

All patients received four courses of intravenous 2-AEH2F (70 mg/kg). Dogs in this sample tolerated IV 2-AEH2F administration (70 mg/kg) well. No changes suggestive of adverse events were reported by owners or noted in physical examination throughout treatment (D1, D8, D15, and D22) or up to D52.

### Laboratory Tests

The mean and standard deviation of laboratory test results obtained on D0, D8, D15, D22, and D52 are shown in Table 2. Lower lymphocyte counts (= 160/μL, >50% below lower limit for age range) suggestive of grade 3 toxicity (adverse event potentially related to test agent) was detected in one dog (patient 7) on D22. Other laboratory changes were limited to grade 2 (adverse event unlikely related to test agent) or grade 1 (adverse event clearly unrelated to test agent). The following laboratory parameters reached abnormal levels over the course the study: hemoglobin levels, leukocyte, lymphocyte and platelet counts, blood glucose and serum ALT and urea levels. Remaining laboratory parameters were within normal ranges according to toxicity criteria adopted in this trial (39) (Table 3).

**Table 2:** Laboratory test results (mean, standard deviation) of dogs treated with 2-AEH2F at different time points.

**Time point**	**Variable**	**Rbc ( × 106/μL)**	**Hb (g/dL)**	**PCV (%)**	**Le (/μL)**	**Ne (/μL)**	**Seg (/μL)**	**Lymph (/μL)**	**Mon (/μL)**	**Eos (/μL)**	**Bas (/μL)**	**Plat (/μL)**	**Gluc (mg/dL)**	**TRI (mg/dL)**	**CHOL (mg/dL)**	**ALT (U/L)**	**AP (U/L)**	**Alb (mg/dL)**	**Urea (mg/dL)**	**Creat (mg/dL)**
D0	mean	7.00	16.64	47.60	8933.00	5817.60	5817.60	2289.40	421.40	333.60	26.00	305000.00	89.71	75.89	235.69	34.33	42.12	3.63	40.53	0.91
D0	SD	0.77	1.54	4.65	3487.06	2127.98	2127.98	1254.25	217.80	214.00	32.39	107642.41	8.18	18.63	54.09	17.57	22.62	0.51	14.51	0.23
D8	mean	6.25	14.95	43.14	9622.00	6485.00	6485.00	2201.00	501.00	434.00	55.00	334000.00	81.00	66.28	222.06	34.71	33.82	3.36	40.59	0.92
D8	SD	0.96	1.64	6.05	2836.21	2143.39	2143.39	786.96	193.65	243.96	47.67	124665.78	18.69	21.62	44.74	23.55	16.52	0.37	16.85	0.23
D15	mean	6.53	15.35	45.50	9849.00	6767.20	6767.20	2142.50	480.90	412.40	45.00	353300.00	82.75	124.22	219.79	36.87	37.05	3.37	37.41	0.89
D15	SD	0.73	1.81	5.54	3716.91	3039.92	3039.92	1099.35	148.08	211.03	43.01	106657.34	12.02	173.11	43.45	29.32	17.19	0.38	19.28	0.26
D22	mean	6.35	15.18	43.90	9742.00	6970.10	6970.10	1836.60	495.50	345.60	48.00	332888.89	87.13	67.29	225.93	40.15	35.69	3.34	37.00	0.92
D22	SD	0.84	2.16	6.12	3223.87	2473.59	2473.59	968.79	134.26	101.42	33.27	86412.45	17.13	22.66	46.15	37.60	15.60	0.45	19.70	0.20
D52	mean	6.96	16.19	46.80	9050.00	6071.70	6386.70	2163.00	350.20	381.50	80.60	282900.00	95.36	83.96	196.36	38.41	28.47	5.26	40.85	0.87
D52	SD	0.85	1.95	5.07	3173.76	2449.71	2802.34	766.97	221.23	225.74	66.96	103748.04	19.04	47.54	78.44	22.76	19.46	6.04	15.60	0.19

*2-AEH2F, 2-Aminoethyl Dihidrogen Phosphate; SD, standard deviation; Rbc, red blood cells; Hb, hemoglobin; PCV, packed cell volume; Le, leukocytes; Ne, neutrophils; Seg, segmented neutrophils; Lymph, lymphocytes; Mon, monocytes; Eos, eosinophils; Bas, basophils; Plat, platelets; Gluc, glucose; TRI, triglycerides; CHOL, cholesterol; ALT, alanine aminotransferase; AP, alkaline phosphatase; Alb, albumin; Creat, creatinine. D0, preoperative period; D8, second 2-Aminoethyl Dihydrogen Phosphate (2-AEH2F) administration; D15, third 2-AEH2F administration; D22, fourth 2-AEH2F administration; D52, 30^th^ day after of 2-AEH2F protocol completion. No dog presented band neutrophils*.

**Table 3:** Number of dogs showing laboratory changes of toxicity grade following treatment with 2-AEH2F (D8 onwards) (39).

**Parameter**	**Toxicity grade 1**	**Toxicity grade 2**	**Toxicity grade 3**
	**Number of dogs (Reference of toxicity)**	**Number of dogs (Reference of toxicity)**	**Number of dogs (Reference of toxicity)**
Hb (g/dL)	1 (R <25% of LLA)	–	–
Le (/μL)	1 (R <25% of LLA)	–	–
Lymph (/μL)	–	3 (R 25 to 50% of LLA)	1 (R > 50% of LLA)
Plat (/μL)	2 (100.000 to LLA)	–	–
Gluc (mg/dL)	6 (ULA, 160 mg/dL)	–	–
ALT (U/L)	1 (ULA to 1.5 × ULA)	1 (1.5 to 4 × ULA)	–
Urea (U/L)	6 (1 to 1.5 × ULA)	1 (1.5 to 3.0 × ULA)	1 (1.5 to 3.0 × ULA)

*2-AEH2F, 2-Aminoethyl Dihidrogen Phosphate; Hb, hemoglobin; Le, leukocytes; Lymph, lymphocytes; Plat, platelets; Gluc, glucose; ALT, alanine aminotransferase; R, reduction; LLA, lower limit for age range; ULA, upper limit for age range; –, unchanged; D8, second 2-2-AEH2F treatment. Toxicity grade 1, adverse event clearly not associated with test agent. Toxicity grade 2, adverse event dubiously associated with test agent. Toxicity grade 3, adverse event potentially associated with test agent*.

### Progression

Tumor recurrence occurred in three out of 10 (33%) dogs in this sample (patients 1, 4, and 6) after D52. Two of these patients (1 and 4) also had MCT metastasis after D52, received chemotherapy with vinblastine or lomustine, and died of MCT-related causes. Patient 6 was reoperated and died of causes unrelated to MCT. Three out of 10 dogs (33%) in this sample died of causes unrelated to MCT, as follows: gastroenteritis (patient 6), skin lymphoma (patient 3), and primary lung cancer (patient 2). Five out of 10 dogs (50%) remain free of recurrence or metastasis to date (Table 4).

**Table 4:** Progression of dogs submitted to treatment with 2-AEH2F.

**Patient**	**Metastasis**	**Recurrence**	**Other ChT after D52**	**Other tumors after D52**	**Death related mast cell tumor**	**Other causes of death**	**TFS (days)**	**OS (days)**
1	Yes–after D52	Yes- after D52	Lomustine	Splenic tumor	Yes	No	163	172
2	No	No	No	Lung câncer	No	Lung cancer	147	154
3	No	No	No	Skin lymphoma	No	Skin lymphoma	65	1,145
4	Yes–after D52	Yes- after D52	Vinblastine	No	Yes	No	270	421
5	Yes–on D0	No	No	No	No	No	368	685
6	No	Yes–after D52	No	No	No	Gastroenteritis	807	807
7	No	No	No	No	No	No	673	673
8	No	No	No	No	No	No	888	888
9	No	No	No	No	No	No	1,375	1,375
10	No	No	No	No	No	No	1,235	1,235

*2-AEH2F, ChT, chemotherapy; TFS, tumor-free survival; OS, overall survival. D0, preoperative period; D52, 30^th^ day after 2-AEH2F protocol completion*.

**Figure 1:**
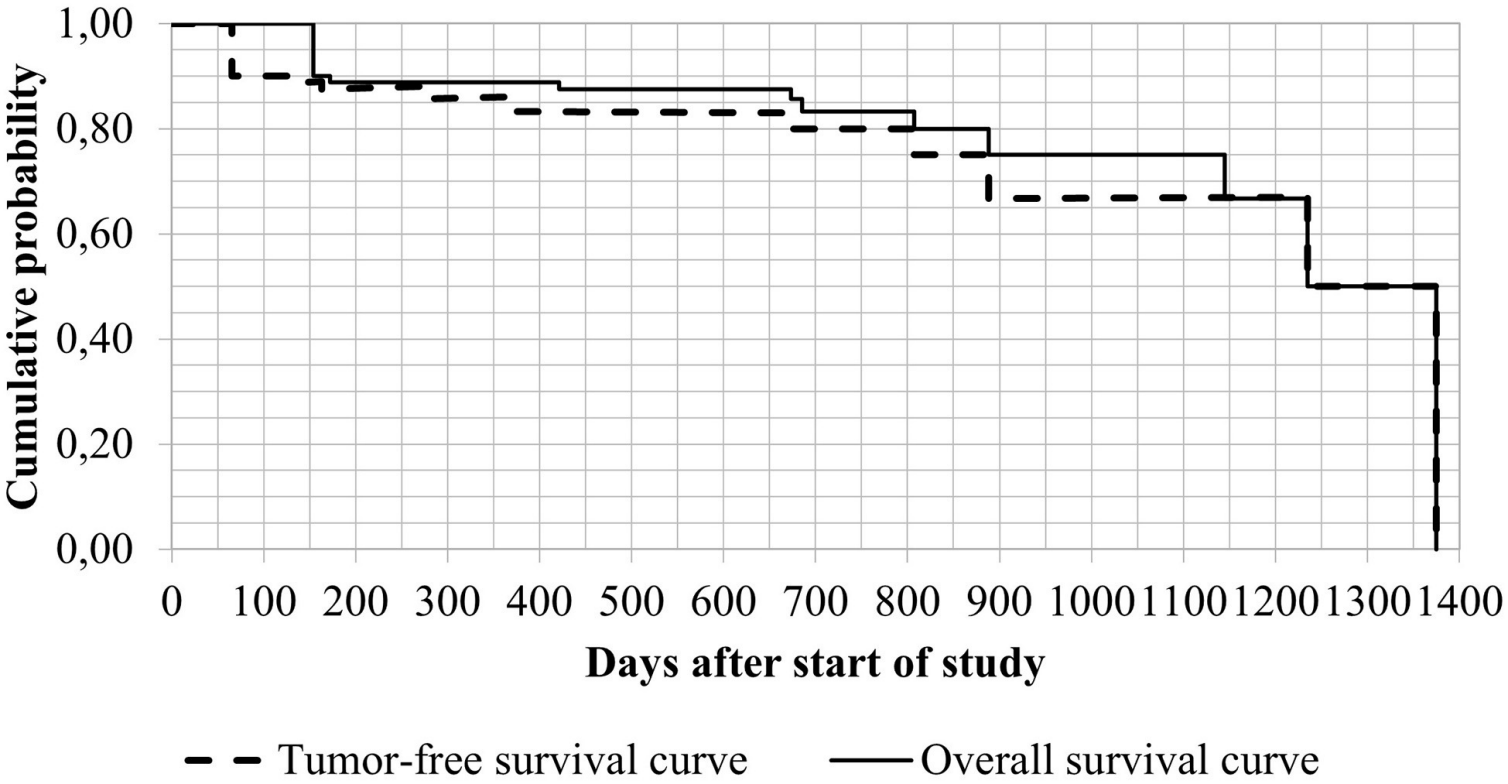
Kaplan-Meier survival curves of patients treated with 2-AEH2F.

The mean TFS and OS corresponded to 599.1 ± 469 and 755.5 ± 423.5 days, respectively (Figure 1).

### Statistics

Laboratory variables other than red blood cell count, hemoglobin level, and platelet count did not differ significantly between time points in this study (Friedman test). However, when the three statistically significant variables were analyzed using the Wilcoxon test, sometime points tended to stand out (*p* values near αBonf = 0.005). As regards confidence intervals and respective means, the mean values of each variable did not vary across time points (Tables 5, 6).

**Table 5:** Complete blood count parameters and respective p values (Friedman and Wilcoxon tests).

**Parameter**	**Rbc**	**Hg**	**PCV**	**Le**	**Ne**	**Seg**	**Lymph**	**Mon**	**Eos**	**Bas**	**Plat**
*N*	10	10	10	10	10	10	10	10	10	10	9
Friedman test (α = 0.05)	0.03*	0.04*	0.09	0.86	0.62	0.71	0.46	0.12	0.74	0.52	0.03*
**Wilcoxon test (α**_**Bonferroni**_ **=** **0.005)**											
D0–D8	0.0051	0.0051	0.0074	0.3900	0.2800	0.2800	0.8780	0.1690	0.2000	0.0280	0.0740
D0–D15	0.1082	0.0592	0.3258	0.8800	0.6500	0.6500	0.3860	0.3860	0.1700	0.2350	0.0280
D0–D22	0.0365	0.0382	0.1374	0.4400	0.1400	0.1400	0.1690	0.2410	0.7200	0.1540	0.0690
D0–D52	0.9188	0.5076	0.6829	0.5100	0.5800	0.5800	0.6460	0.3860	0.4800	0.0500	0.8780
D8–D15	0.3329	0.5076	0.2859	0.5800	0.5800	0.5800	0.3330	0.7990	0.8600	0.8780	0.6830
D8–D22	0.7596	0.6465	0.6458	0.8800	0.2800	0.2800	0.0740	1.0000	0.6400	0.6740	0.7670
D8–D52	0.0929	0.2026	0.1530	0.3900	0.4800	0.5800	0.6460	0.0280	0.4400	0.2840	0.1390
D15–D22	0.2127	0.7211	0.1118	0.8000	0.5100	0.5100	0.7210	0.5070	0.6100	0.9060	0.5150
D15–D52	0.1258	0.2023	0.5376	0.3300	0.3300	0.3300	0.5080	0.0830	0.5700	0.1680	0.0130
D22–D52	0.0741	0.1688	0.2583	0.5100	0.1100	0.4400	0.2410	0.0250	0.3900	0.1250	0.0280

*n, number of evaluated dogs; Rbc, red blood cells; Hb, hemoglobin; PCV, packed cell volume; Le, leukocytes; Ne, neutrophils; Band, band neutrophils; Seg, segmented neutrophils; Lymph, lymphocytes; Mon, monocytes; Eos, eosinophils; Bas, basophils; Plat, platelets; NA, not assessed; D0, preoperative period; D8, second 2-Aminoethyl Dihidrogen Phosphate (2-AEH2F) administration; D15, third 2-AEH2F administration; D22, fourth 2-AEH2F administration; D52, 30^th^ day after of 2-AEH2F protocol completion*.

*Significant differences between values.

**Table 6:** Biochemical parameters and respective p values (Friedman and Wilcoxon tests).

**Parameter**	**Gluc**	**TRI**	**CHOL**	**ALT**	**AP**	**Alb**	**Urea**	**Creat**
*n*	6	6	6	10	10	10	10	10
α = 0.05								
Friedman test (α = 0.05)	0.35	0.91	0.62	0.96	0.19	0.06	0.71	0.52
**Wilcoxon test (*****α***_**Bonferroni**_ **=0.005)**								
D0–D8	0.2250	0.6700	0.3300	0.6500	0.0470	0.0093	0.5800	0.5940
D0–D15	0.3700	1.0000	0.0500	0.5800	0.3330	0.0593	0.3300	0.5750
D0–D22	0.4630	0.8700	0.4000	0.9600	0.4450	0.0166	0.3300	0.7790
D0–D52	0.7350	0.4800	0.2600	0.8000	0.0220	0.3329	0.9600	0.2840
D8–D15	0.2370	0.7200	0.9600	0.9500	0.8780	0.7987	0.8000	0.9590
D8–D22	0.1760	0.6800	0.6800	0.7200	0.9590	0.9594	0.4400	0.8780
D8–D52	0.0120	0.5100	0.1400	0.7200	0.0830	0.7213	0.7200	0.1130
D15–D22	0.6240	0.8600	0.6800	0.6500	0.4450	0.8782	1.0000	1.0000
D15–D52	0.0690	0.9500	0.5100	0.5800	0.1690	0.9594	0.2800	0.4840
D22–D52	0.8890	0.6700	0.7800	0.7200	0.2410	0.9594	0.2400	0.0920

*n, number of evaluated dogs; Gluc, glucose; TRI, triglycerides; CHOL, cholesterol; ALT, alanine aminotransferase; AP, alkaline phosphatase; Alb, albumin; Creat, creatinine; D0, preoperative period; D8, second 2-Aminoethyl Dihidrogen Phosphate (2-AEH2F) administration; D15, third 2-AEH2F administration; D22, fourth 2-AEH2F administration; D52, 30th day after of 2-AEH2F protocol completion.*

*Significant differences between values.

## Discussion

To the knowledge of the authors, this is the first study on the use of 2-AEH2F in dogs with MCT and has demonstrated its safety in the species, but low efficacy in MCT with poor immunohistochemical prognostic factors.

In this research, weekly intravenous administration of 2-AEH2F solution at 70 mg/kg doses did not induce adverse events or relevant laboratory changes in treated patients. Gastrointestinal signs and fever associated with chemotherapeutic agents such as vinblastine and lomustine (21, 24, 40–42) were therefore not observed in dogs treated with 2-AEH2F. Also, these dogs did not require ancillary therapy with H2 antagonists, as is often the case in patients undergoing chemotherapy (41).

Laboratory parameter values were not significantly affected by therapy with 2-AEH2F in this study. In contrast, neutropenia, sepsis, liver toxicity and azotemia have been widely reported following treatment with drugs such as vinblastine and lomustine (21, 24, 40–43). Despite significant differences in red blood cell count, hemoglobin level and platelet count (Friedman test), it was not possible to determine when these differences occurred (Wilcoxon test), even though *p* values tended to near 0.005 (α_Bonf)_ at some time points (Tables 5, 6). Small sample size may have interfered with this analysis. Laboratory toxicity grade in dogs in this sample was not correlated with the target agent (grades 1 and 2 in most cases, with only one dog presenting with grade 3 lymphocytic toxicity or uremia) (39) (Tables 2, 3). Among the indices that reached grade 3 toxicity in two patients, lymphopenia may be related to the stress situation and uremia alone may reflect dehydration. Therefore, red blood cell count, hemoglobin level and platelet count differences were not clinically relevant.

Antineoplastic phospholipids have been shown to affect cellular cholesterol efflux (42, 43). The mechanism involves structural modifications to the ATP-binding cassette transporter 1 (ABCA1) that mediates the unidirectional efflux of cellular phospholipid to lipid-poor apolipoprotein A-I and other apolipoproteins (44). Still, the mean and the median serum total cholesterol and triglyceride levels remained unchanged in patients treated with 2-AEH2F in this research.

In this study, metastasis and tumor recurrence were documented in 2/10 and 3/10 of dogs treated with 2-AEH2F, respectively. However, two out of three dogs presenting with recurrence were the same dogs who had metastasis (i.e., no more than 3/10 of patients had tumor recurrence overall). Of patients presenting with MCT metastasis or recurrence after D52, three had pattern II or III in KIT stain evaluation and two had ki67 expression over 23%. These findings are thought to be correlated with poor prognostic factors (15, 18) (Table 1) and may have interfered with the effects of 2-AEH2F treatment on TFS in these cases. In remaining patients, KIT staining pattern and Ki67 expression patterns were not indicative of poor prognosis (pattern I and <23%, respectively) (15, 18). Lack of metastasis or recurrence in these patients may therefore not have reflected therapeutic effects of 2-AEH2F. In this study two out of three dogs that developed recurrence or metastasis had grade II (low grade) tumors. Therefore, histological classification of MCT into two or three grades (according to cell differentiation) did not correlate with TFS or OS, as previously reported (6, 45, 46). However, these patients had compromised surgical margins. Low tumor recurrence rates (7%) have been reported in patients with compromised surgical margins submitted to adjuvant therapy with vinblastine and prednisone (40). In this study, 2-AEH2F was not able to prevent tumor cell dissemination in cases with positive margins.

Half (50%; *n* = 5) of dogs treated with 2-AEH2F in this trial went into remission and remain in remission to date. This remission rate is higher than rates reported in dogs treated with lomustine alone or a combination of vinblastine and lomustine (5 and 25%, respectively) (43), but lower than rates reported following treatment with vinblastine alone (71.4%) (41). Tumor free survival of dogs treated with 2-AEH2F in this study (755 days; minimum of 154 and maximum of 1,375 days) (Figure 1) was longer compared to TFS reported following adjuvant treatment with lomustine (122; 42–347 days) (21), vinblastine and prednisone (304; 37–1,964 days, 322 days and 241.5; 10–1,521 days) (40, 41, 44) or toceranib and prednisone (159; 20–990 days) (44). As in this study, a different one reported TFS of 211 days in dogs developing metastasis following surgical resection and treatment with a combination of vinblastine and prednisone, whereas those not developing metastasis after similar treatment achieved TFS of 757 days (41). Tumor-free survival and tumor remission in this study was longer or similar to TFS data reported in literature. However, the lack of poor prognostic factors (Ki67 <23% and KIT staining pattern I or II) (15, 18) in patients achieving complete remission in this sample may have contributed to longer survival. Longer TFS has been recently reported in dogs with free surgical margins compared to dogs with compromised surgical margins submitted to treatment with vinblastine (893; 70–893 days and 181; 70–548 days, respectively) (20).

Development of new tumors unrelated to MCT after D52 (skin lymphoma and primary lung cancer, respectively) in two dogs suggests 2-AEH2F did not prevent other neoplastic conditions in these patients (Table 4). New tumor growth has also been reported in dogs submitted to chemotherapy with lomustine (24).

The small sample size is a limitation of this study. Also, lack of studies investigating serum 2-AEH2F levels and urinalysis in treated patients and pharmacokinetic aspects of this drug precluded more accurate characterization of the product. Finally, the fact that not all tumors were amenable to immunohistochemical analysis may have interfered with findings in some cases.

In conclusion, intravenous administration of 2-AEH2F at doses and intervals described in this study was thought to be safe in dogs. However, treatment with 2-AEH2F alone was not deemed sufficiently effective to prevent tumor recurrence, new tumor growth or metastasis in cases of canine MCT with poor immunohistochemical prognostic factors. Further studies are warranted to support the use of 2-AEH2F for canine MCT, considering the high safety levels and ease of administration observed in this trial. Based on our results, 2-AEH2F can be investigated as a treatment in association with other forms of therapy for MCT or other types of tumors in canine species.

## Data Availability Statement

The raw data supporting the conclusions of this article will be made available by the authors, without undue reservation.

## Ethics Statement

The animal study was reviewed and approved by Ethics Committee on Animal Use of the School of Veterinary Medicine and Animal Science of University of São Paulo (São Paulo - Brazil). Protocol number 9825270116. Written informed consent was obtained from the owners for the participation of their animals in this study.

## Author Contributions

EJ, SM, DM, and JM conceived the study and designed the overall study design. EJ analyzed the data and wrote the paper with support of all authors. EJ and SM diagnosed and treated the dogs and provided all relevant data. AA conducted the anesthetic procedures. JM conducted the laboratory tests. CL conducted the ultrasound exams. BC performed the immunohistochemical tests. HS performed the statistical analysis. All authors critically revised the manuscript, read and approved the final manuscript.

## Funding

This study was partially funded by *Coordenação de Aperfeiçoamento de Pessoal de N*í*vel Superior - Brasil* (CAPES) - Finance Code 001.

## Conflict of Interest

The authors declare that the research was conducted in the absence of any commercial or financial relationships that could be construed as a potential conflict of interest.

## Publisher's Note

All claims expressed in this article are solely those of the authors and do not necessarily represent those of their affiliated organizations, or those of the publisher, the editors and the reviewers. Any product that may be evaluated in this article, or claim that may be made by its manufacturer, is not guaranteed or endorsed by the publisher.

## Abbreviations

2-AEH2F: 2-Aminoethyl Dihydrogen Phosphate
ALT: Alanine aminotransferase
AP: Alkaline phosphatase
HPLC: High performance liquid chromatography
MCT: Canine mast cell tumors
OS: overall survival
PE: Phosphoethanolamine
TFS: tumor-free survival.
